# Intratumoral heterogeneity as a source of discordance in breast cancer biomarker classification

**DOI:** 10.1186/s13058-016-0725-1

**Published:** 2016-06-28

**Authors:** Emma H. Allott, Joseph Geradts, Xuezheng Sun, Stephanie M. Cohen, Gary R. Zirpoli, Thaer Khoury, Wiam Bshara, Mengjie Chen, Mark E. Sherman, Julie R. Palmer, Christine B. Ambrosone, Andrew F. Olshan, Melissa A. Troester

**Affiliations:** Lineberger Comprehensive Cancer Center, University of North Carolina at Chapel Hill, Chapel Hill, NC USA; Department of Epidemiology, University of North Carolina at Chapel Hill, 135 Dauer Drive, CB 7435, Chapel Hill, NC 27599 USA; Department of Pathology, Brigham & Women’s Hospital, Boston, MA USA; Department of Cancer Prevention and Control, Roswell Park Cancer Institute, Buffalo, NY USA; Department of Pathology, Roswell Park Cancer Institute, Buffalo, NY USA; Department of Biostatistics, University of North Carolina at Chapel Hill, Chapel Hill, NC USA; Department of Genetics, University of North Carolina at Chapel Hill, Chapel Hill, NC USA; Division of Cancer Prevention, National Cancer Institute, Bethesda, MD USA; Slone Epidemiology Center, Boston University, Boston, MA USA

**Keywords:** Automated algorithm, Digital pathology, Discordance, Estrogen receptor, HER2, Immunohistochemistry, Intratumoral heterogeneity, Progesterone receptor, Tissue microarray

## Abstract

**Background:**

Spatial heterogeneity in biomarker expression may impact breast cancer classification. The aims of this study were to estimate the frequency of spatial heterogeneity in biomarker expression within tumors, to identify technical and biological factors contributing to spatial heterogeneity, and to examine the impact of discordant biomarker status within tumors on clinical record agreement.

**Methods:**

Tissue microarrays (TMAs) were constructed using two to four cores (1.0 mm) for each of 1085 invasive breast cancers from the Carolina Breast Cancer Study, which is part of the AMBER Consortium. Immunohistochemical staining for estrogen receptor (ER), progesterone receptor (PR), and human epidermal growth factor receptor 2 (HER2) was quantified using automated digital imaging analysis. The biomarker status for each core and for each case was assigned using clinical thresholds. Cases with core-to-core biomarker discordance were manually reviewed to distinguish intratumoral biomarker heterogeneity from misclassification of biomarker status by the automated algorithm. The impact of core-to-core biomarker discordance on case-level agreement between TMAs and the clinical record was evaluated.

**Results:**

On the basis of automated analysis, discordant biomarker status between TMA cores occurred in 9 %, 16 %, and 18 % of cases for ER, PR, and HER2, respectively. Misclassification of benign epithelium and/or ductal carcinoma in situ as invasive carcinoma by the automated algorithm was implicated in discordance among cores. However, manual review of discordant cases confirmed spatial heterogeneity as a source of discordant biomarker status between cores in 2 %, 7 %, and 8 % of cases for ER, PR, and HER2, respectively. Overall, agreement between TMA and clinical record was high for ER (94 %), PR (89 %), and HER2 (88 %), but it was reduced in cases with core-to-core discordance (agreement 70 % for ER, 61 % for PR, and 57 % for HER2).

**Conclusions:**

Intratumoral biomarker heterogeneity may impact breast cancer classification accuracy, with implications for clinical management. Both manually confirmed biomarker heterogeneity and misclassification of biomarker status by automated image analysis contribute to discordant biomarker status between TMA cores. Given that manually confirmed heterogeneity is uncommon (<10 % of cases), large studies are needed to study the impact of heterogeneous biomarker expression on breast cancer classification and outcomes.

**Electronic supplementary material:**

The online version of this article (doi:10.1186/s13058-016-0725-1) contains supplementary material, which is available to authorized users.

## Background

Heterogeneity in biomarker expression between tumors is the basis for breast cancer subtyping and precision medicine [1]. However, intratumoral heterogeneity, often reflecting spatial heterogeneity of biomarker expression within a single tumor, has important implications for accurate tumor classification, and it may impact both epidemiologic research [2] and clinical decision-making [3].

Approximately 10–20 % of tumors are found to have disagreement in estrogen receptor (ER), progesterone receptor (PR), and human epidermal growth factor receptor 2 (HER2) status upon repeat assay, as assessed by studies examining interlaboratory agreement rates [4–6]. A variety of technical factors contribute to lack of interlaboratory agreement, including differences in antibody or assay type; level of laboratory experience; and tumor sampling, fixation, and storage protocols [4, 7–14]. In addition to these technical factors, repeat assays are commonly carried out using a separate tumor block and therefore may test a different area of the tumor, suggesting that spatial heterogeneity of biomarker expression may also contribute to discordance [15]. However, the frequency and sources of intratumoral ER, PR, and HER2 heterogeneity have not been evaluated in population-based studies.

Using tissue microarrays (TMAs) comprising two to four tumor cores for each of 1085 cases from the Carolina Breast Cancer Study (CBCS) in the African American Breast Cancer Epidemiology and Risk (AMBER) Consortium, we identified cases with core-to-core discordance in ER, PR, and HER2 status using automated digital image analysis. Discordant cases were manually reviewed to identify technical and biological factors contributing to variability in biomarker expression. We estimated the frequency of intratumoral ER, PR, and HER2 heterogeneity among biomarker-positive cases and evaluated the impact of biomarker discordance on case-level ER, PR, and HER2 status agreement between TMAs and the clinical record.

## Methods

### Study population

The AMBER Consortium, comprising the Black Women’s Health Study, the Women’s Circle of Health Study, the Multi-Ethnic Cohort Study, and the CBCS, was formed to identify genetic and nongenetic factors associated with specific breast cancer subtypes [16]. Standardization of staining and scoring protocols for classification of invasive breast cancer subtypes is a major objective of this collaborative study [17]. For our present analyses, we used phase III of CBCS, a population-based, case-only study conducted in North Carolina between 2008 and 2013 [18]. The study was approved by the Office of Human Research Ethics at the University of North Carolina at Chapel Hill, and written informed consent was obtained from each participant.

Clinical ER, PR, and HER2 status was abstracted from medical records. Cases noted in the medical records to have weak or borderline ER and PR expression were classified as ER-positive and PR-positive, respectively, according to current guidelines [19]. Paraffin-embedded tumor blocks were requested from participating pathology laboratories for each case, and study pathologists marked hematoxylin and eosin (H&E)-stained slides to indicate areas enriched for invasive breast cancer for coring. TMAs were constructed from 1.0-mm cores, and these comprised 1238 invasive breast cancer cases (*n* = 600 African American and *n* = 638 non-African American). Sections from the top and bottom of each TMA block were stained with H&E and reviewed by study pathologists to ensure that only TMA cores with top and bottom tumor in addition to sufficient tumor cellularity (≥50 tumor cells per core) were included in our analysis. We excluded cases that were missing clinical ER, PR, or HER2 status (*n* = 76), as well as cases represented by only one evaluable core on our TMAs (*n* = 66) as core-to-core discordance could not be assessed. Finally, we excluded cases with cores derived from multiple tumor blocks (from either single or multiple tumors; *n* = 11), leaving us with 1085 cases included in the present analysis. A comparison of CBCS phase III cases on TMAs (41 % of all cases in CBCS phase III) with those not on TMAs showed no differences with respect to race or clinical ER, PR, or HER2 status. However, phase III cases on TMAs were older and more likely to be postmenopausal, and they had higher combined grade but lower stage and smaller tumor size.

### Classification of central ER, PR, and HER2 status using tissue microarrays

Detailed methods for immunohistochemical (IHC) staining of ER, PR, and HER2 in CBCS have been described elsewhere [17]. Of the 1085 cases included in the present analysis, 685 cases (63 %) had 4 cores, 287 cases (27 %) had 3 cores, and 113 cases (10 %) had 2 cores for ER. The distribution of numbers of cores per case was similar for PR and HER2. Automated digital image analysis of IHC staining was performed using a Genie classifier (Aperio Technologies, Vista, CA, USA) and the Nuclear v9 algorithm (for ER and PR) or Membrane v9 algorithm (for HER2) (Aperio Technologies, Vista, CA, USA); this analysis is described in more detail in our previous publication [17]. Core-to-case collapsing to assign case-level biomarker status was carried out using a tumor cellularity-weighted approach, as previously described [17]. Briefly, the weighted average of percent positivity was calculated by summing the product of percent positivity and core weight across all cores per case. Core weight was defined as the number of tumor nuclei in a given core divided by the total number of tumor nuclei across all cores for that case. A 1 % threshold for ER and PR positivity [19] was subsequently applied to define dichotomous positive/negative case-level status for ER and PR. Case-level HER2 status was defined as positive (3+; ≥10 % of tumor cells staining at the 3+ intensity level), equivocal (2+; <10 % of tumor cells staining at the 3+ intensity level and ≥10 % of tumor cells staining at the 2+ intensity level), or negative (0/1+; all other cases). We reported previously that these automated scoring methods showed very high agreement with manual review by study pathologists and with the clinical record [17].

### Identification of technical and biological sources of ER, PR, and HER2 discordance

Discordant cases (i.e., cases with discordant biomarker status between TMA cores) were manually reviewed by a breast pathologist (JG) to identify those in which discordance was caused by spatial heterogeneity of biomarker expression and those in which discordance was caused by misclassification by the automated algorithm. We restricted this manual evaluation to discordant cases with positive case-level biomarker status (≥1 %), because biomarker discordance between cores in negative cases was due to random variation around the 1 % threshold used to define case status. Only one ER-negative case showed >10 % variation in ER status between cores (Additional file 1: Figure S1). Manual assessment of intratumoral HER2 heterogeneity was performed for discordant cases with at least one 3+ core, since heterogeneity within HER2-negative cases (i.e., cases with only 0/1+ and 2+ cores) is less clinically relevant.

### Impact of core-to-core discordance in biomarker status on case-level agreement with the clinical record

We identified cases with discordant biomarker status between cores using dichotomous ER and PR status (i.e., <1 %, ≥1 %) and three-category HER2 status (i.e., 0/1+, 2+, 3+). We also explored ER and PR discordance using a 10 % threshold. The frequency of discordance did not differ by race for any biomarker (data not shown). Median tumor cellularity was compared between cases with discordant versus concordant biomarker status between cores using rank-sum tests, and chi-square tests were used to compare rates of biomarker discordance among cases with two, three, and four TMA cores. Cohen’s kappa statistics were used to evaluate agreement between clinical and central TMA classifications for ER, PR, and HER2 [20] overall and stratified by concordant/discordant status between TMA cores. Statistical analyses were conducted using STATA version 13.1 software (StataCorp, College Station, TX, USA).

## Results

### Frequency of intratumoral ER, PR, and HER2 heterogeneity

**Fig. 1:**
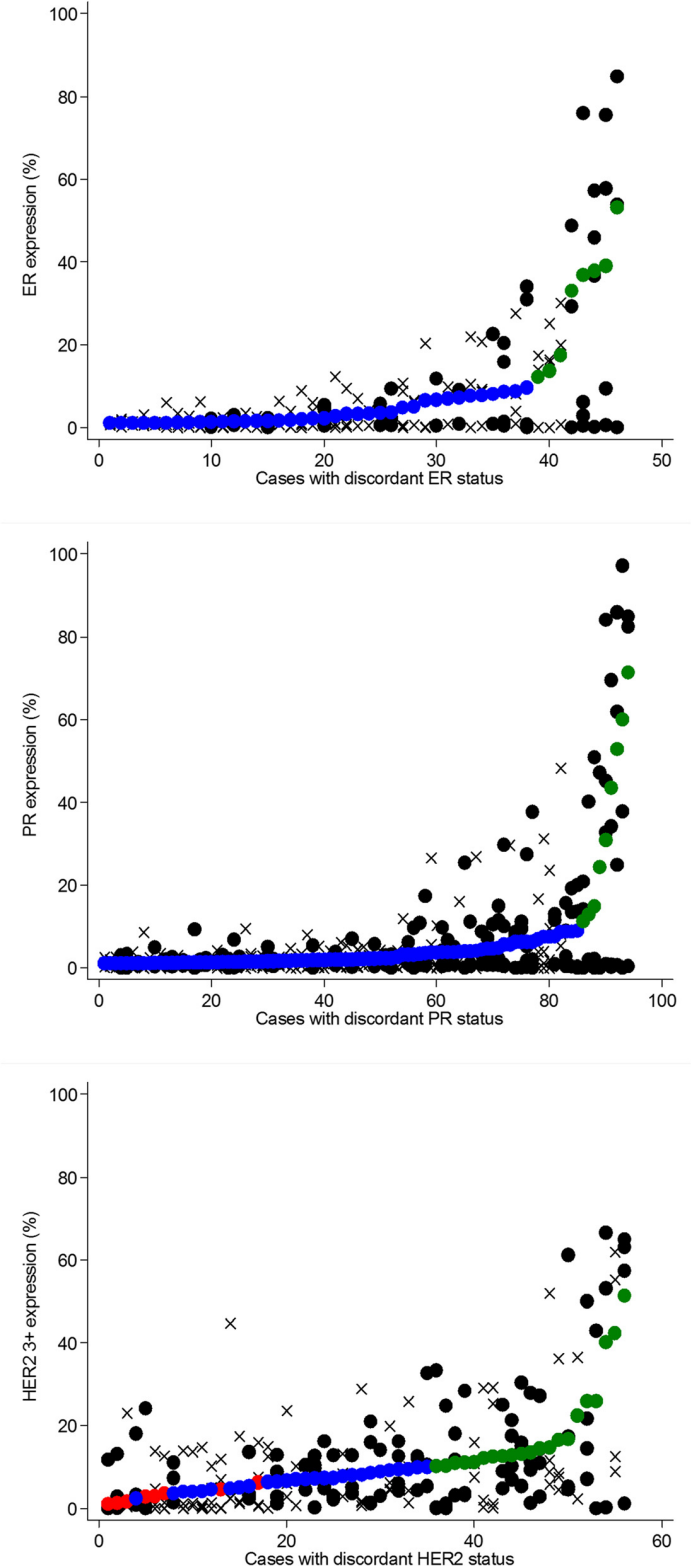
ER, PR, and HER2 expression in cases with discordant biomarker status between cores, restricted to positive (≥1 %) cases for ER and PR and to cases with at least one 3+ core (≥10 % 3+) for HER2. Individual cases are ordered on the *x*-axis by case-level biomarker expression level (*smaller solid circles*: *red* = negative, *blue* = borderline/equivocal, *green* = positive). Individual cores for each case are represented by *solid black circles* for cases with manually confirmed heterogeneity or by *X*’s for cases without manually confirmed heterogeneity. *ER* estrogen receptor, *HER2* human epidermal growth receptor 2, *PR* progesterone receptor

**Fig. 2:**
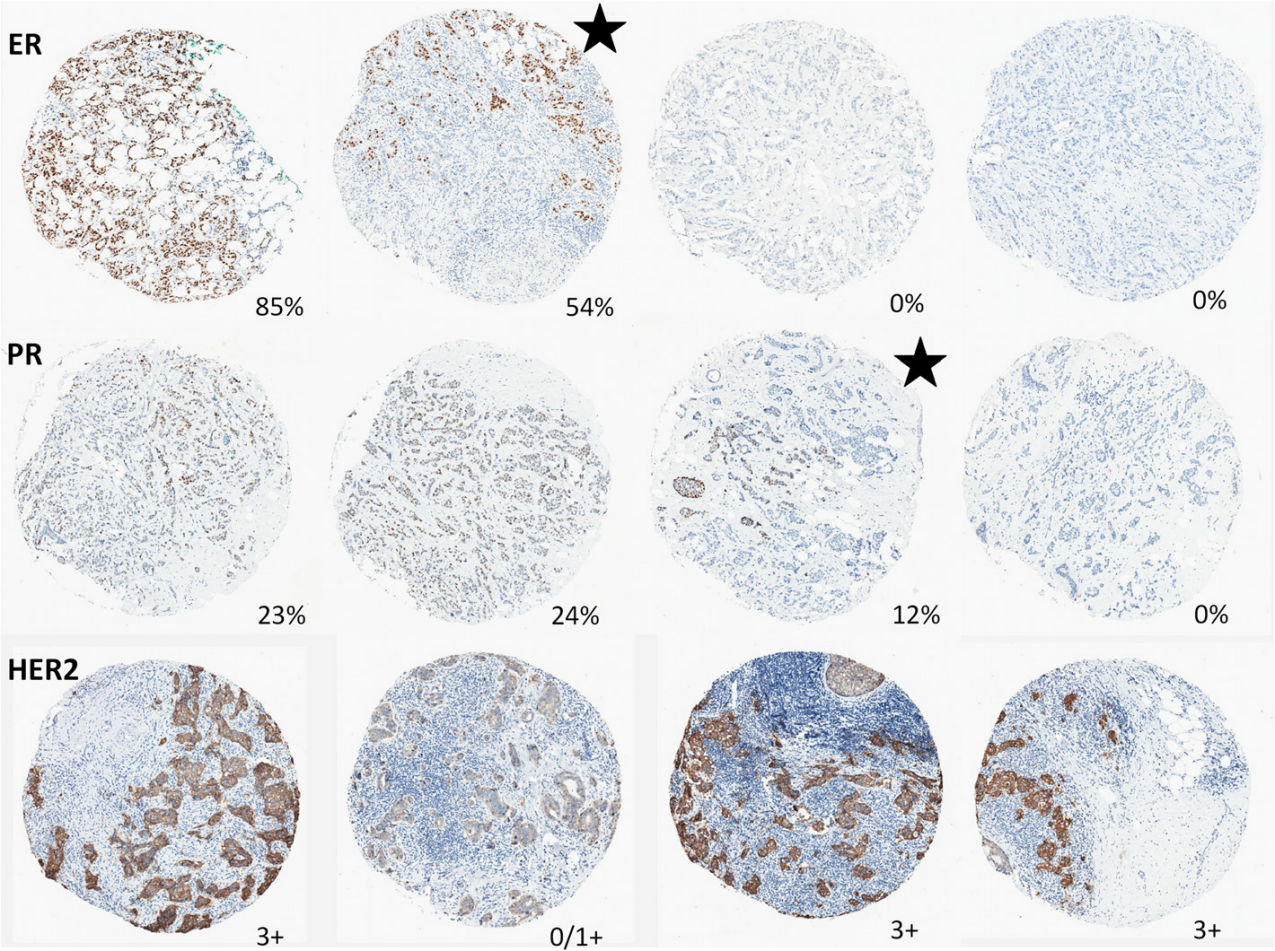
Representative images of cases with manually confirmed heterogeneous expression of ER, PR, and HER2 between any two cores from the same case. The percentage of ER- and PR-positive cells or HER2 status is indicated for each core. The *starred* cores illustrate examples of intracore heterogeneity. *ER* estrogen receptor, *HER2* human epidermal growth receptor 2, *PR* progesterone receptor

**Fig. 3:**
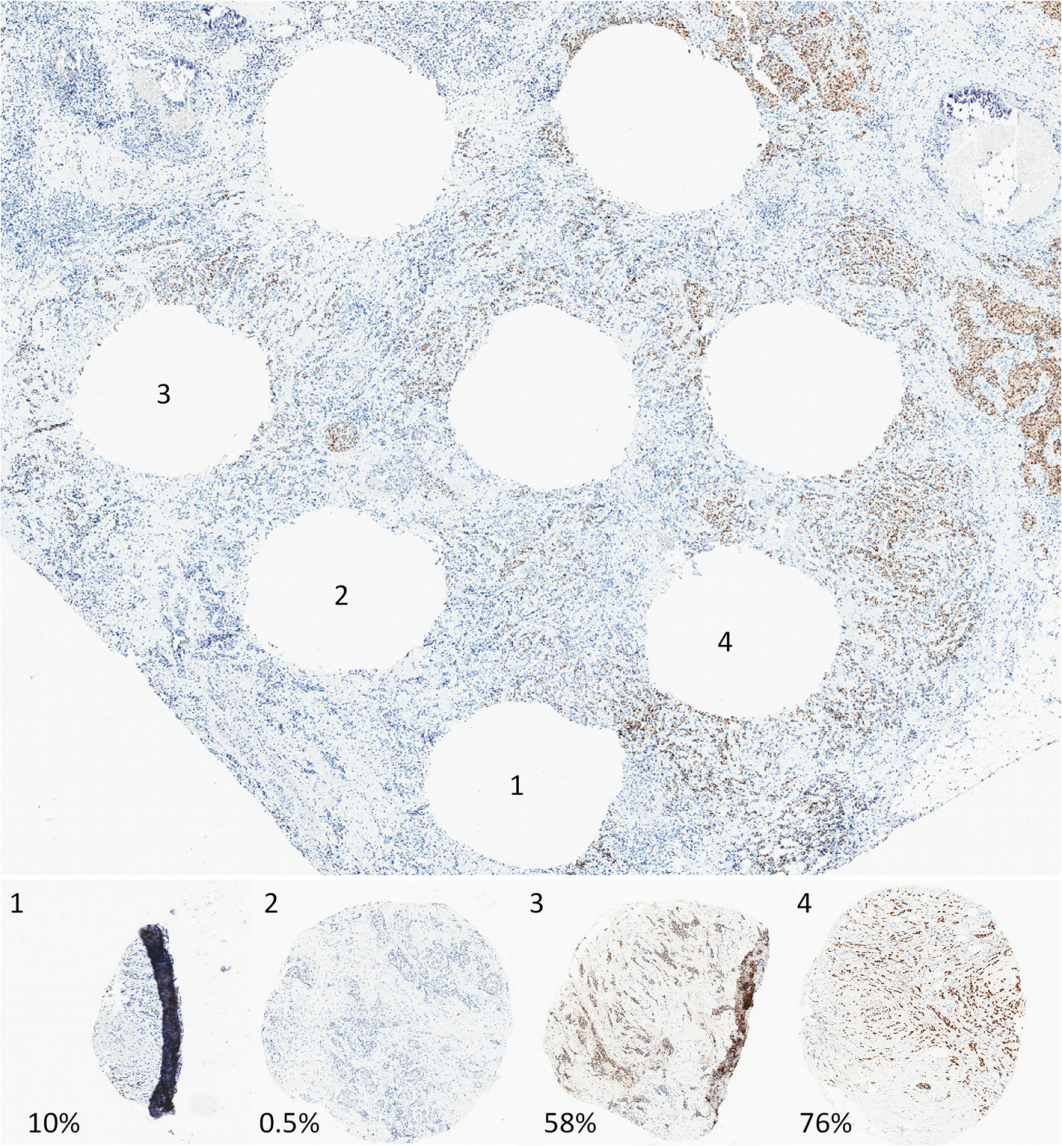
Representative images of ER staining in four cores and the tissue section from which they were removed, in a case with manually confirmed heterogeneous ER expression. Note the variability in staining across the whole tissue section, which is reflected in the variable expression levels in the four cores. *ER* estrogen receptor

Among 1085 cases of invasive breast cancer, cases with discordant biomarker status between TMA cores numbered 100 (9 %) for ER, 169 (16 %) for PR, and (18 %) for HER2. We conducted a manual review of all discordant biomarker-positive cases (46 discordant ER-positive cases, 94 discordant PR-positive cases, and 56 discordant HER2 cases with at least one positive [3+] core). Figure 1 shows core- and case-level biomarker expression levels for manually reviewed cases, with individual cases represented on the *x*-axis and biomarker expression shown on the *y*-axis. Cores from cases with manually confirmed heterogeneity are denoted with a *solid black circle*, while all other cores are denoted with an X. Among discordant ER-positive cases, 16 (35 % of manually reviewed cases and 2 % of all 784 ER-positive cases) were manually confirmed to be spatially heterogeneous. Of these 16 cases with manually confirmed ER heterogeneity, 7 had negative (<1 %) and borderline (≥1 to <10 %) cores (i.e., no positive [≥10 %] cores) and 9 had both negative (<1 %) and positive (≥10 %) cores. The frequency of PR heterogeneity was higher than that of ER, with 53 (56 % of manually reviewed cases and 7 % of all 739 PR-positive cases) manually confirmed to be heterogeneous. Of the 53 cases with manually confirmed PR heterogeneity, 30 had negative (<1 %) and borderline (≥1 to < 10 %) cores only, while 23 had both negative (<1 %) and positive (≥10 %) cores. Representative images of ER and PR heterogeneity are shown in Figs. 2 and 3.

HER2 heterogeneity was manually confirmed in 31 cases (55 % of manually reviewed cases, and 21 % of all 148 cases with at least one 3+ core). A representative image of HER2 heterogeneity is shown in Fig. 2. Of these 31 cases with spatially heterogeneous HER2 expression, 19 were comprised of both 2+ and 3+ cores (i.e., no 0/1+ cores); 7 were comprised of both 0/1+ and 3+ cores (i.e., no 2+ cores); and 5 were comprised of 0/1+, 2+, and 3+ cores. When equivocal cores were excluded and only cases with both negative (0/1+) and positive (3+) cores were classified as heterogeneous (*n* = 12), the frequency of HER2 heterogeneity was similar to that of PR (21 % of manually reviewed cases, and 8 % of all cases with at least one 3+ core). Very few cases had simultaneous manually confirmed heterogeneity of multiple biomarkers; one case had heterogeneous expression of both ER and PR, and five cases had heterogeneous expression of both PR and HER2.

### Identification of confounding factors producing spurious biomarker heterogeneity

**Fig. 4:**
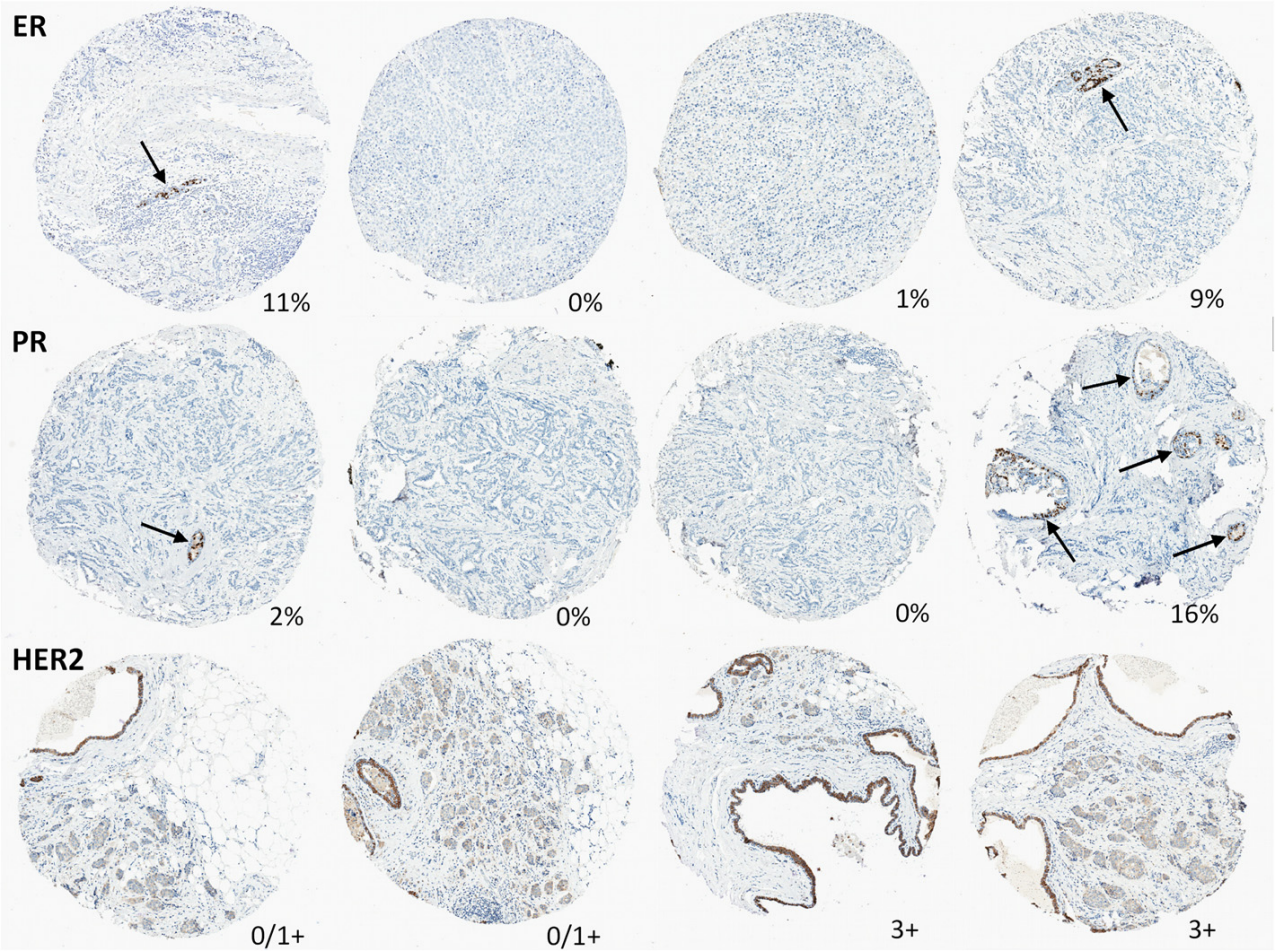
Representative images of cases with discordant ER, PR, and HER2 status between any two cores from the same case due to presence of benign epithelium (*arrows* for ER, PR) and clinging ductal carcinoma in situ (HER2). Percentage of ER- and PR-positive cells or HER2 status is indicated for each core. *ER* estrogen receptor, *HER2* human epidermal growth receptor 2, *PR* progesterone receptor

Manual review of discordant biomarker-positive cases revealed that, in some cases, automated algorithms detected discordance between cores due to admixed benign epithelium and/or ductal carcinoma in situ (DCIS) (Fig. 4). Admixture of biomarker-positive DCIS in a background of biomarker-negative invasive carcinoma was particularly relevant in the assessment of HER2 heterogeneity. Various types of technical artefacts (such as foreign material or cytoplasmic staining) also led to false-positive automated scores. In some cases with lower tumor cellularity, the automated image analysis algorithm underestimated the number of biomarker-negative cells, producing falsely elevated expression levels. However, this source of technical error affected only tumors with expression levels that were very close to the threshold used to define biomarker status.

### Impact of tumor sampling on frequency of ER, PR, and HER2 discordance

We next sought to identify factors that could be used to identify cases with increased likelihood of manually confirmed intratumoral heterogeneity. To identify causes of biomarker discordance and to identify criteria for prioritizing manual review, we focused on all cases with discordant biomarker status between cores and not just on manually reviewed cases. We found that cases with discordant ER and PR status between cores had significantly lower median tumor cellularity, but that tumor cellularity was similar in cases with concordant and discordant HER2 status (Table 1). The frequency of discordant biomarker status between cores was not significantly impacted by the number of TMA cores per case for ER or PR. HER2 discordance rates differed somewhat by number of cores per case, but there was no clear pattern of association (Table 1).

**Table 1:** Tumor sampling characteristics of cases with estrogen receptor, progesterone receptor, and human epidermal growth factor receptor 2 discordance between tissue microarray cores in phase III of the Carolina Breast Cancer Study

	ER				PR				HER2			
	*n* (%)	Conc (*n* = 985)	Disc (*n* = 100)	*p* Value	*n* (%)	Conc (*n* = 916)	Disc (*n* = 169)	*p* Value	*n* (%)	Conc (*n* = 889)	Disc (*n* = 196)	*p* Value
Cellularity, median (IQR)	1085 (100)	5225 (2846–8869)	3564 (2142–6074)	<0.001	1085 (100)	5772 (3018–9851)	3785 (2238–7925)	<0.001	1,085 (100)	7303 (4134–11,329)	8233 (4414–12,675)	0.282
Core number												
2	113 (10)	102 (90)	11 (10)	0.978	113 (10)	111 (98)	10 (9)	0.098	106 (10)	87 (82)	19 (18)	0.043
3	287 (26)	261 (91)	26 (9)		299 (28)	253 (85)	46 (15)		289 (27)	223 (77)	66 (23)	
4	685 (63)	622 (91)	63 (9)		673 (61)	560 (83)	113 (17)		690 (64)	579 (84)	111 (16)	

Conc concordant biomarker status across all cores for a given case, Disc discordant biomarker status between any two cores for a given case, *ER* estrogen receptor, *HER2* human epidermal growth factor receptor 2, *PR* progesterone receptor

### Impact of discordant biomarker status between cores on agreement with the clinical record

Overall, agreement between central TMAs and the clinical record was substantial for ER (94 %) and PR (89 %; Table 2). However, ER and PR agreement with the clinical record was lower among cases with discordant ER and PR status between cores (70 % and 61 %, respectively). Conversely, clinical record agreement was very high in cases with concordant ER and PR status across all TMA cores for a given case (96 % and 95 %, respectively; Table 2). For HER2, overall agreement was high (88 %), but was reduced among cases with discordant HER2 status (57 %). When analysis was restricted to cases with concordant HER2 status across all TMA cores, agreement was similar to that for cases with concordant ER and PR status (96 %; Table 2).

**Table 2:** Impact of discordant estrogen receptor, progesterone receptor, and human epidermal growth factor receptor 2 status between cores on agreement between tissue microarrays and the clinical record in phase III of the Carolina Breast Cancer Study

Central TMA				Agreement (%)	Kappa (95 % CI)
Clinical ER status	ER-negative, *n* (%)		ER-positive, *n* (%)		
All cases, *n* = 1085					
ER-negative, *n* (%)	259 (90)		42 (5)	94	0.84 (0.80–0.87)
ER-positive, *n* (%)	28 (10)		756 (95)		
Concordant, *n* = 985					
ER-negative, *n* (%)	217 (96)		32 (4)	96	0.89 (0.86–0.92)
ER-positive, *n* (%)	8 (4)		728 (96)		
Discordant, *n* = 100					
ER-negative, *n* (%)	42 (68)		10 (26)	70	0.39 (0.22–0.57)
ER-positive, *n* (%)	20 (32)		28 (74)		
Clinical PR status	PR-negative, *n* (%)		PR-positive, *n* (%)		
All cases, *n* = 1085					
PR-negative, *n* (%)	305 (80)		41 (6)	89	0.76 (0.72–0.80)
PR-positive, *n* (%)	75 (20)		664 (94)		
Concordant, *n* = 916					
PR-negative, *n* (%)	246 (91)		27 (4)	95	0.87 (0.83–0.90)
PR-positive, *n* (%)	23 (9)		620 (96)		
Discordant, *n* = 169					
PR-negative, *n* (%)	59 (53)		14 (24)	61	0.25 (0.12–0.38)
PR-positive, *n* (%)	52 (47)		44 (76)		
Clinical HER2 status	Negative	Equivocal	Positive		
All cases, *n* = 1085					
Negative, *n* (%)	864 (93)	16 (80)	28 (21)	88	0.57 (0.51–0.63)
Equivocal, *n* (%)	49 (5)	2 (10)	13 (10)		
Positive, *n* (%)	17 (2)	2 (10)	94 (70)		
Concordant, *n* = 889					
Negative, *n* (%)	768 (99)	7 (88)	22 (21)	96	0.78 (0.72–0.84)
Equivocal, *n* (%)	4 (0)	0 (0)	1 (1)		
Positive, *n* (%)	5 (1)	1 (12)	81 (78)		
Discordant, *n* = 196					
Negative, *n* (%)	96 (63)	9 (75)	6 (19)	57	0.16 (0.07–0.28)
Equivocal, *n* (%)	45 (29)	2 (17)	12 (39)		
Positive, *n* (%)	12 (8)	1 (8)	13 (42)		

*Abbreviations: ER* estrogen receptor, *HER2* human epidermal growth factor receptor 2, *PR* progesterone receptor, *TMA* tissue microarray

Concordant cases are those with the same biomarker status across all cores for a given case, while discordant cases are those with discordant biomarker status between any two cores for a given case

## Discussion

Intratumoral biomarker heterogeneity may pose a challenge for accurate classification of breast cancer, with implications both for clinical decision making and for epidemiologic research. However, the frequency and sources of intratumoral ER, PR, and HER2 heterogeneity have not been well-characterized, particularly in population-based studies. Using TMAs comprising multiple cores per case, we observed that cases with discordant biomarker status between cores by automated digital image analysis had reduced agreement with the clinical record. Manual review of discordant cases revealed that 35–56 % of discordant biomarker status between cores was caused by spatially heterogeneous expression of ER, PR, and HER2, which was observed in 2 %, 7 %, and 8 % of all biomarker-positive cases, respectively.

Our findings demonstrate that automated algorithms cannot reliably distinguish between IHC-stained tumor and nontumor cells. Therefore, admixture of tumor and DCIS and/or benign epithelium can potentially lead to tumor biomarker misclassification by automated analysis if biomarker status is discordant between tumor and nontumor tissues. Synchronous DCIS and invasive cancers typically share tumor characteristics and hormone receptor status [21]. However, HER2-positive DCIS within an HER2-negative invasive tumor has been observed [22], and this may pose a challenge for the use of digital algorithms to properly classify the HER2 status of invasive carcinomas. In addition, admixed benign epithelium, which often expresses both ER and PR, can produce false positivity in hormone receptor-negative tumors. However, we previously showed that computing average biomarker expression across cores after weighting cores by tumor cellularity diminishes the influence of small discordant regions and produces high agreement (≥88 % for all biomarkers) with the clinical record.

Intratumoral ER heterogeneity has previously been suggested to be a rare phenomenon [23], although the frequency in a population-based setting has not been established. Using an automated approach to identify cases with discordant ER status between cores, followed by manual review, we observed intratumoral heterogeneity of ER expression in 2 % of all ER-positive cases. These results are consistent with prior studies suggesting that the frequency of intratumoral ER heterogeneity ranges from 0.5 % to 10 % [23–26]. It has been hypothesized that some intratumoral heterogeneity could be technical in origin, arising from inadequate sample fixation, and this may contribute to the higher heterogeneity rates reported by some studies. However, differential rates of heterogeneity across different biomarkers in our study and the tiny minority of samples with simultaneous heterogeneity of more than one biomarker suggest that this may be an unlikely explanation for our findings. We also show that inadequate tumor sampling may contribute to biomarker discordance, as tumors with low cellularity were more likely to have discordant ER and PR status between cores. This finding supports our previous research in the AMBER Consortium showing that ER and PR agreement rates between TMAs and the clinical record were reduced in cases with low tumor cellularity [17]. Our frequency estimate for intratumoral PR heterogeneity (7 % of PR-positive cases) appears lower than that reported previously (approximately 20 % in two studies [23, 24]). However, one of these prior studies used whole-tissue slides from a consecutive series of patients with breast cancer treated in a tertiary care facility [23], while the other examined agreement between core needle biopsy and surgical specimens in women presenting with a palpable mass [24]. As such, in contrast to our present analysis, these prior studies likely overrepresent a more aggressive set of cancers. If heterogeneity is associated with tumor aggressiveness as hypothesized, this could contribute to differences in frequency across studies.

We observed two types of intratumoral HER2 heterogeneity. Cases with equivocal and positive cores formed the majority, comprising 21 % of cases with at least one HER2-positive core, while only 8 % of cases with at least one positive core also had at least one negative core. A prior study reported the presence of both negative and positive HER2 regions in only 1 % of 921 cases [27], while others reported similar or even lower rates of intratumoral HER2 heterogeneity using IHC analysis [22, 28]. Researchers in several studies have also reported very low rates of heterogeneity of HER2 amplification status using in situ hybridization techniques [27–29]. However, in these prior studies, researchers reported the frequency of HER2 heterogeneity among all cases, and not just among those with areas of HER2 positivity (defined by the presence of at least one positive core in our study). If we had included all cases in our denominator, only 1 % of all cases would have had both positive and negative HER2 cores, in line with prior studies [22, 27, 28].

Tumors with spatially distinct areas of high and low biomarker expression levels may suggest a pattern of heterogeneity referred to as *segregated heterogeneity* [30]. Segregated heterogeneity may be particularly clinically relevant because antiestrogen or HER2-directed therapy may apply a selective pressure for outgrowth of areas lacking the molecular target, with consequences for the subtype for subsequent disease recurrence [31, 32]. Studies of recurrent tumors, particularly those with a subtype distinct from the primary tumor, may be important for understanding the consequences of intratumoral heterogeneity. Similarly, longitudinal studies with quantitative histology and well-characterized spatial biomarker patterns may help improve understanding of the impact of intratumoral heterogeneity on breast cancer outcomes. If intratumoral heterogeneity proves to be a poor prognostic feature as theorized, identification of demographic and tumor characteristics associated with intratumoral heterogeneity could help to identify patients who may benefit from more extensive tumor workup and, potentially, more aggressive therapy. This work is currently underway in the AMBER Consortium.

This study should be considered in light of some limitations. First, the tumor specimens used for clinical workup may have been biopsy specimens or separate blocks from those used to construct central TMAs, and therefore it is possible that the clinical record and the central results represent distinct tumor regions. However, different origins of tumor specimens would be a random source of error, unlikely to bias our findings away from the null. Second, even multiple 1.0-mm TMA cores represent only a small portion of the entire tumor, and therefore it is possible that we underestimated the frequency of intratumoral heterogeneity in the present study. However, our rates of intratumoral heterogeneity are similar to those reported previously. Finally, due to tumor sampling at the time of breast cancer surgery, we were unable to assess temporal intratumoral heterogeneity in this study. Despite the theoretical importance of temporal heterogeneity [32], spatial heterogeneity at the time of tumor excision is arguably the most relevant for clinical management of breast cancer.

These limitations are balanced by several important strengths. Since automated staining of TMAs is becoming more widely used [17, 33], we assessed automated evidence of intratumoral heterogeneity (i.e., biomarker discordance between TMA cores), and our results can therefore be used to guide manual review. Our automated image analysis methods are well-validated and produce very high agreement with manual scoring of TMAs in CBCS [17]. The analysis of the population-based CBCS ensured excellent representation of both African American and non-African American cases in this study, and we were able to infer that race does not strongly influence rates of intratumoral heterogeneity. In addition, procurement of tissue from multiple clinical centers, representing community-based and referral centers, ensured that our study was not biased toward more aggressive cancers commonly seen in referral centers. Given that clinical biomarker status was measured at multiple different laboratories and according to multiple protocols, the substantial rates of agreement between central TMA results and the clinical record provide reassurance that ER, PR, and HER2 staining are well-standardized across clinical care settings.

## Conclusions

Our findings demonstrate that the presence of admixed benign epithelium and/or DCIS in TMA cores can cause biomarker misclassification when using automated methods to quantify IHC staining. However, manually confirmed intratumoral heterogeneity accounted for approximately half of all cases with core-to-core discordance in biomarker status on TMAs. These results suggest that intratumoral heterogeneity may contribute to discordance in ER, PR, and HER2 status, with possible implications for breast cancer subtype classification. The low frequency of intratumoral heterogeneity underscores the robustness of ER and HER2 for guiding targeted treatment. Future work, likely with large studies or consortia, is required to identify risk factors for intratumoral heterogeneity and to determine its impact on treatment response.

## Abbreviations

AMBER Consortium, African American Breast Cancer Epidemiology and Risk Consortium; CBCS, Carolina Breast Cancer Study; Conc, concordant; DCIS, ductal carcinoma in situ; Disc, discordant; ER, estrogen receptor; H&E, hematoxylin and eosin; HER2, human epidermal growth factor receptor 2; IHC, immunohistochemical; PR, progesterone receptor; TMA, tissue microarray

## Additional file

Additional file 1: ER and PR expression levels among cases with discordant biomarker status between cores, restricting to cases with negative (<1 %) ER and PR expression. Cases are ordered on the X-axis by case-level positivity status. Individual cores are represented by black crosses, and case-level positivity status is represented by red crosses. (TIF 452 kb)
