# Supplementary figures and images for: Intratumoral heterogeneity as a source of discordance in breast cancer biomarker classification
Source: Breast Cancer Res. 2016 Jun 28;18:68. doi: 10.1186/s13058-016-0725-1 (PMC4924300; doi:10.1186/s13058-016-0725-1)

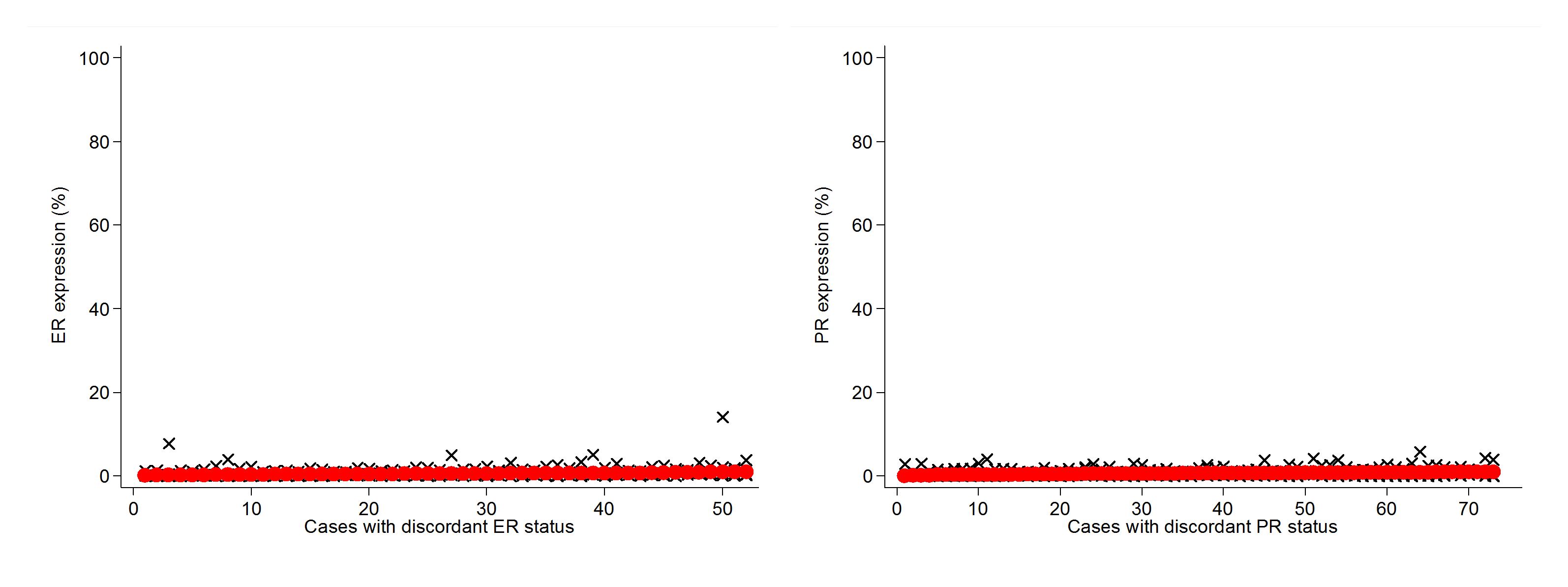

Supplement: Additional file 1: — ER and PR expression levels among cases with discordant biomarker status between cores, restricting to cases with negative (<1 %) ER and PR expression. Cases are ordered on the X-axis by case-level positivity status. Individual cores are represented by black crosses, and case-level positivity status is represented by red crosses. (TIF 452 kb) [file 13058_2016_725_MOESM1_ESM.tif]
